# Hepatocyte growth factor is a prognostic marker in patients with colorectal cancer: a meta-analysis

**DOI:** 10.18632/oncotarget.15589

**Published:** 2017-02-21

**Authors:** Chao-yuan Huang, Qian-yi Zhou, Yue Hu, Yi Wen, Zhen-wen Qiu, Man-guang Liang, Jun-ling Mo, Jian-hua Xu, Cong Sun, Feng-bin Liu, Xin-lin Chen

**Affiliations:** ^1^ The First Clinical College, Guangzhou University of Chinese Medicine, Guangzhou, China; ^2^ School of Basic Medical Science, Guangzhou University of Chinese Medicine, Guangzhou, China; ^3^ The First Affiliated Hospital, Guangzhou University of Chinese Medicine, Guangzhou, China; ^4^ The Second Clinical College, Guangzhou University of Chinese Medicine, Guangzhou, China; ^5^ The Second Affiliated Hospital, Guangzhou University of Chinese Medicine, Guangdong, China; ^6^ Zhongshan School of Medicine, Sun Yat-Sen University, Guangdong, China

**Keywords:** hepatocyte growth factor, prognosis, marker, colorectal cancer, meta-analysis

## Abstract

Hepatocyte growth factor (HGF) is a crucial factor associated with development, progression and metastasis of colorectal cancer (CRC). However, its prognostic value remains unclear. Thus studies referring to the correlation between HGF and CRC patients’ prognosis were included to explore the role of HGF in CRC. At last nine articles were included. The results showed that the over-expression of HGF was associated with a poor prognosis, presented through overall survival (OS, Hazard ratio (HR) = 2.50, 95% confidence interval (CI): 2.12–2.96) and disease-free survival (DFS, HR = 1.99, 95% CI: 1.59–2.50). Subgroup analysis indicated that no significant difference was found between the Asian countries (OS: HR = 2.37; DFS: HR = 2.02) and the non-Asian countries (OS: HR = 3.15; DFS: HR = 1.87), between the studies that used univariate analyses (OS: HR = 2.51; DFS: HR = 2.07) and those that used multivariate analyses (OS: HR = 2.65; DFS: HR = 1.78), and between metastatic CRC (OS: HR = 2.26; DFS: HR = 2.06) and stage I-IV CRC (OS: HR = 3.08; DFS: HR = 0.70). Our meta-analysis has shown that the over-expression of HGF is valuable in CRC prognosis evaluation. This conclusion should be further confirmed by large-sample cohort studies.

## INTRODUCTION

Colorectal cancer (CRC, including colon cancer and rectal cancer) is the third most commonly diagnosed cancer in males and the second in females, with an estimated 1.4 million cases and 693,900 deaths in 2012. Moreover, the incidence has continued to increase in certain countries where historically, the risk has been low [1]. In 2015, it was projected that 132,700 (69,090 men and 63,610 women) individuals would be newly diagnosed with colorectal cancer, which accounted for a prevalence rate of 8% in both sexes [2]. Almost 694,000 deaths from CRC are estimated to have occurred in 2012, and these accounted for approximately 8.5% of all cancer deaths [3]. With the ever increasing incidence and mortality, CRC is highly regarded as a clinical problem.

Hepatocyte growth factor (HGF, DFNB39, HPTA), which is also called scatter factor, is a gene primarily involved in the regulation of cell growth, motility and morphogenesis [4]. HGF induces complex intracellular signaling networks, which results in cell proliferation and cell survival, and leads to regeneration and homeostasis of various types of epithelial tissues [5]. HGF exerts its biological effects via its tyrosine kinase cell surface receptor, hepatocyte growth factor receptor (HGFR), which is also known as mesenchymal to epithelial transition factor (MET) [6, 7]. When it is aberrantly activated, the HGF-MET pathway is involved in the regulation of proliferation, motility, invasion and metastasis via the phosphorylation and activation of downstream signaling pathways, which consequently, promote tumorigenesis [8].

The expression of HGF plays a critical role in cell proliferation and is involved in CRC [9]. Conclusions from published studies on the correlation between HGF and the survival time of patients with CRC were, for the most part, consistent except for the study conducted by Karabulut, which found a negative correlation between HGF expression and the survival [10]. Studies that have revealed the pathogenic roles of HGF in CRC are not rare, but evidence-based medicine has not verified the prognosis or the survival time of CRC patients in the context of HGF expression. Whether high HGF expression leads to a poor prognosis of CRC remains inconclusive.

Therefore, we systematically evaluated the correlation between HGF and the prognosis and survival of CRC patients and provided clinical guidance for the treatment of CRC.

## RESULTS

### Search results and study characteristics

**Figure 1:**
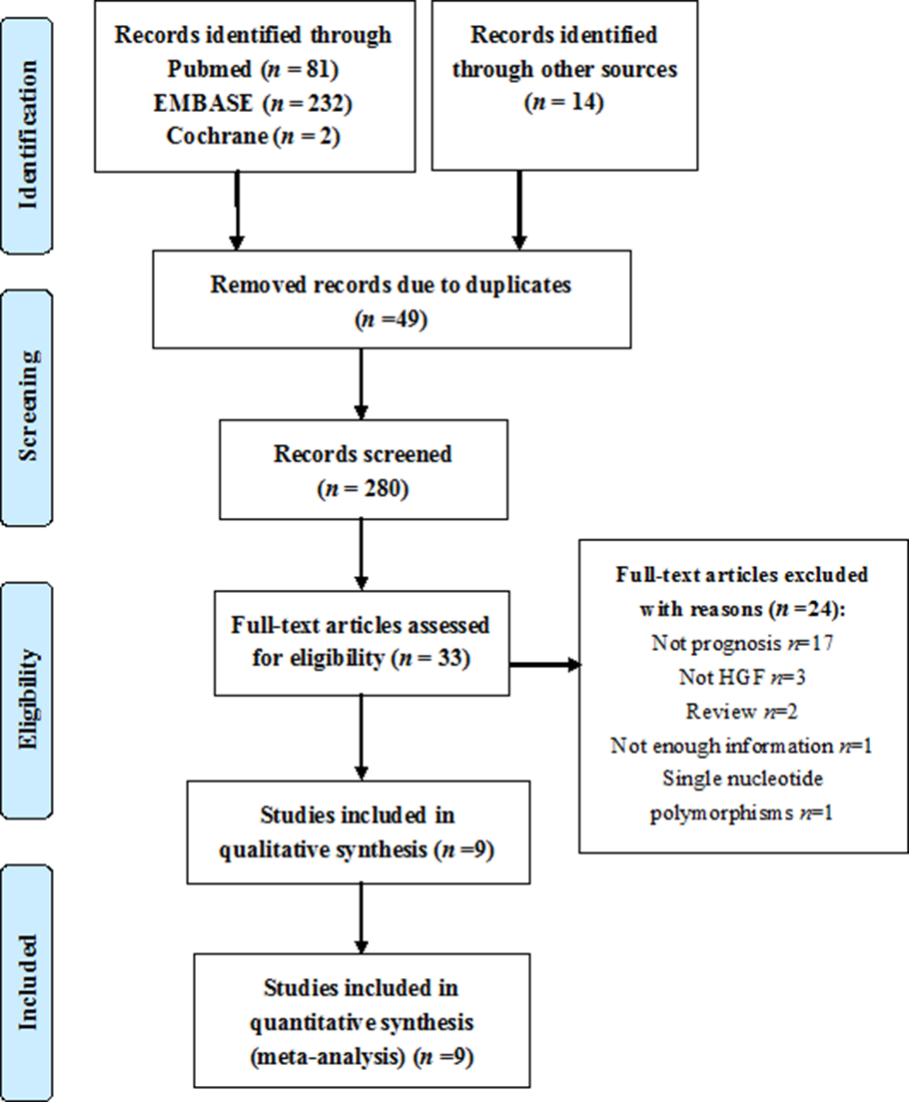
Flow chart of the search strategy

The study searches were performed as shown in Figure 1. At first, 329 studies were obtained from multiple databases, and then, 49 duplicates were removed. Of the relevant studies, 247 studies were excluded based on the title and abstract. Subsequently, 33 studies were assessed by screening the full-text, among which 24 articles were excluded. Eventually, nine studies were included in the meta-analysis [5, 10–17].

All the studies were published from 2004 to 2016, with incremental tendency since 2015. Most of the studies were conducted in developed countries, such as the USA, Germany, Netherlands, Japan and Turkey. Data on patients in six studies were collected from 1991 to 2014, while other studies did not mention the collection time [5, 13, 17]. Altogether, the nine studies included 777 patients, and each study contained samples that ranged from 18 to 184 patients. The proportion of males was approximately 50–70% in all studies except for the study performed by Kammula (36.5%) [16]. The age of the included patients ranged from 24 to 95 years. Four studies enrolled patients with metastatic CRC (mCRC), while others enrolled patients with stage I-IV CRC. Among the nine studies, five used univariate analyses [5, 10, 12, 13, 17], and four used multivariate analyses [11, 14–16]. The cut-off value of HGF ranged from 0.204 to 4.0 ng/ml in most of the included studies.

Eight studies reported the prognostic value of HGF with respect to overall survival (OS) in patients with CRC [5, 10–16]. Among them, four directly reported hazard ratio (HR) [11, 12, 14, 16], while the others provided survival curves (Table 1) [5, 10, 13, 15]. Seven of the eight studies found that the over-expression of HGF was an indicator for poor prognosis and poor OS, while the remaining study revealed no statistical significance between HGF over-expression and OS [10]. Four studies reported the prognostic value of HGF with respect to disease-free survival (DFS) in patients with CRC [10–12, 17]. Of the four studies, 2 studies directly reported HR, while the others reported survival curves [11, 12].

**Table 1:** The main characteristics and results of the included studies

Author	Year of publication	Country	Time	Sample size	Male (%)	Mean age (range)	Patients	Stage I+II (%)	Surgical therapy (%)	Chemotherapy(%)	Radiotherapy (%)	Follow-up (median, months)	High expression (%)	Cut-off value (ng/ml)	Test sample	Test content	Test method	Analytic method	Outcome	HR for OS	95% CI HR for OS	HR for DFS	95% CI HR for DFS
Takahashi N	2014	Japan	2008-2011	103	63.11	62 (26-81)	mCRC	0	0	NR	0	NR	50.49	1.99	Serum	Protein	ELISA	Multivariate	OS, DFS	2.24	(1.34–3.74)	1.78	(1.14–2.78)
Seneviratne D	2015	American	NR	78	58.97	NR (45-95)	CRC	34.6	NR	NR	NR	NR	18	NR	Tissue	Protein	IHC	Univariate	OS	4.23*	(2.59–6.91)	NR	NR
Yonesaka K	2015	Japan	2010-2012	51	62.75	62 (41-85)	mCRC	0	0	NR	0	NR	49.02	0.204	Plasma	Protein	ELISA	Univariate	OS, DFS	2.23	(1.78–2.80)	2.12	(1.61–2.79)
Hinz S	2015	Germany	NR	18	61.11	66 (48-78)	mCRC	0	NR	55.6	0	50	55.56	4	Serum	Protein	ELISA	Univariate	OS	3.41*	(1.06–11.00)	NR	NR
Toiyama Y	2009	Japan	1996-2002	184	60.33	64 (27-86)	CRC	51.6	100	48.4	NR	65	36.41	0.37	Serum	Protein	ELISA	Multivariate	OS	3.1	(1.73–5.54)	NR	NR
Miki C	2006	Japan	1996-1998	147	60.54	66	CRC	53.7	100	46.3	NR	68.4	57.82	0.35	Serum	Protein	ELISA	Multivariate	OS	2.97*	(1.56–5.66)	NR	NR
Kammula US	2007	USA	1991-1993	60	36.51	NR	Colon cancer	41.7	100	NR	NR	73.2	45	NR	Tissue	RNA	PCR	Multivariate	OS	2.44	(1.05–5.68)	NR	NR
de Jong KP	2004	Netherlands	NR	33	60.61	56	mCRC	0	NR	NR	NR	≥24.0	NR	0.4	Blood	Protein	ELISA	Univariate	DFS	NR	NR	5.68*	(1.12–28.88)
Karabulut M	2016	Turkey	2011-2014	103	69.9	60 (24-84)	CRC	45.6	NR	NR	NR	14	NR	3.36	Serum	Protein	ELISA	Univariate	OS, DFS	0.70*	(0.20–2.50)	0.70*	(0.20–2.50)

mCRC: metastatic colorectal cancer; CRC: colorectal cancer; OS: overall survival; DFS: disease-free survival; ELISA: enzyme linked immunosorbent assay; PCR: polymerase chain reaction; IHC: immunohistochemistry; Multivariate survival analyses; Univariate: survial analyses.

* the data was analyzed survival curve: NR: reported.

### Outcomes of the meta-analysis

**Figure 2:**
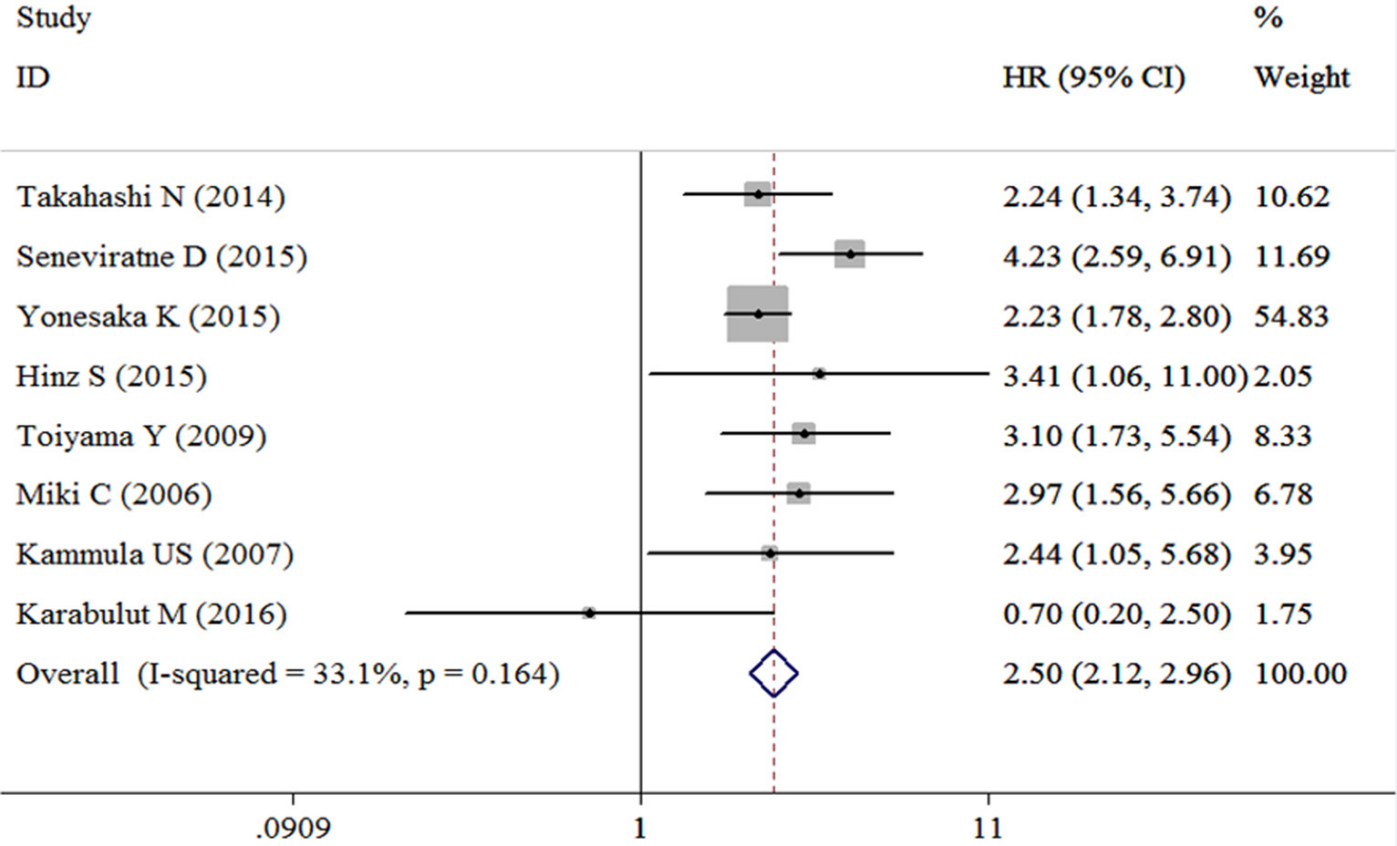
Forest plot evaluating the combined HR between HGF and OS

**Figure 3:**
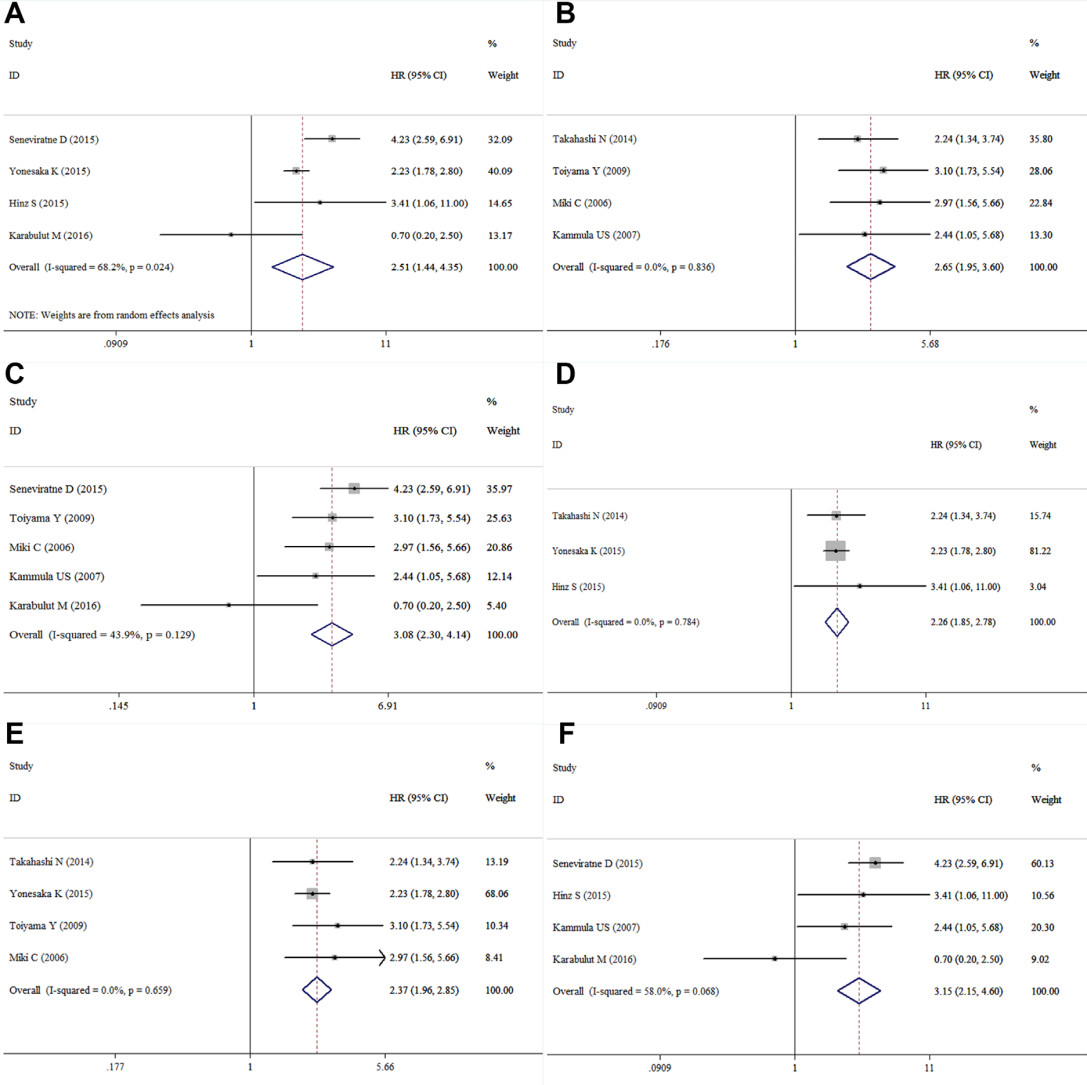
Forest plot of the subgroup analysis of HGF and OS ((**A)**: univariate analysis; (**B**): multivariate analysis; (**C**): Asian countries; (**D**): non-Asian countries; (**E**): I-IV CRC patients; (**F**): mCRC patients).

Eight studies containing the data of HGF and OS in CRC patients were included in this research. The combined HR for the over-expression of HGF on OS was 2.50 (95% confidence interval [CI]: 2.12–2.96); it was based on an analysis under fixed effects model (inconsistency index [I^2^] = 33.1%, χ^2^ = 10.47, *P* = 0.164) (Table 2, Figure 2). Random effect model was adopted in studies performing univariate analysis, and in result the adjusted combined HR was 2.51 (95% CI: 1.44–4.35) (Figure 3A). Fixed effect model was adopted in studies performing multivariate analysis (I^2^ = 0.0%, χ^2^ = 0.86 *P* = 0.836), and the combined HR was 2.65 (95% CI: 1.95–3.60) (Figure 3B) and no heterogeneity was observed. Subgroup analyses on patient categories presented that the combined HR of stage I-IV CRC and mCRC were homogenous, and under fixed effect model, the combined HR of stage I-IV CRC patients and mCRC patients were 3.08 (95% CI: 2.30–4.14) (Figure 3C) and 2.26 (95% CI: 1.85–2.78) (Figure 3D), respectively. The studies conducted in Asia presented a homogeneous result (I^2^ = 0.0%, χ^2^ = 1.60, *P* = 0.659) and the combined HR was 2.37 (95% CI: 1.96–2.85) (Figure 3E). The combined HR of the studies conducted outside from Asia was 3.15 (95% CI: 2.15–4.60) (Figure 3F).

**Table 2:** The results of the meta-analysis

	Number of studies	Patients	HR (95% CI)	Heterogeneity		
				I^2^	χ^2^	*P*
**Overall survival**						
**All**	8	744	2.50 (2.12–2.96)	33.1%	10.47	0.164
Univariate analysis	4	250	2.51 (1.44–4.35)*	68.2%	9.43	0.024
Multivariate analysis	4	494	2.65 (1.95–3.60)	0.0%	0.86	0.836
**Patients**						
I–IV CRC	5	572	3.08 (2.30–4.14)	43.9%	7.14	0.129
mCRC	3	172	2.26 (1.85–2.78)	0.0%	0.49	0.784
**Country**						
Asian	4	485	2.37 (1.96–2.85)	0.0%	1.60	0.659
Non-Asian	4	259	3.15 (2.15–4.60)	58.0%	7.14	0.068
**Disease-free survival**						
**All**	4	290	1.99 (1.59–2.50)	35.1%	4.62	0.201
Univariate analysis	3	187	2.07 (1.59–2.70)	53.4%	4.30	0.117
Multivariate analysis	1	103	1.78 (1.14–2.78)	-	-	-
**Patients**						
I-IV CRC	1	103	0.70 (0.20–2.50)	-	-	-
mCRC	3	187	2.06 (1.64–2.60)	0.0%	1.94	0.378
**Country**						
Asian	2	154	2.02 (1.60–2.55)	0.0%	0.42	0.515
Non-Asian	2	136	1.87 (0.24–14.44)*	74.6%	3.94	0.047

^*^Results based on a random effects model. -: not applicable.

**Figure 4:**
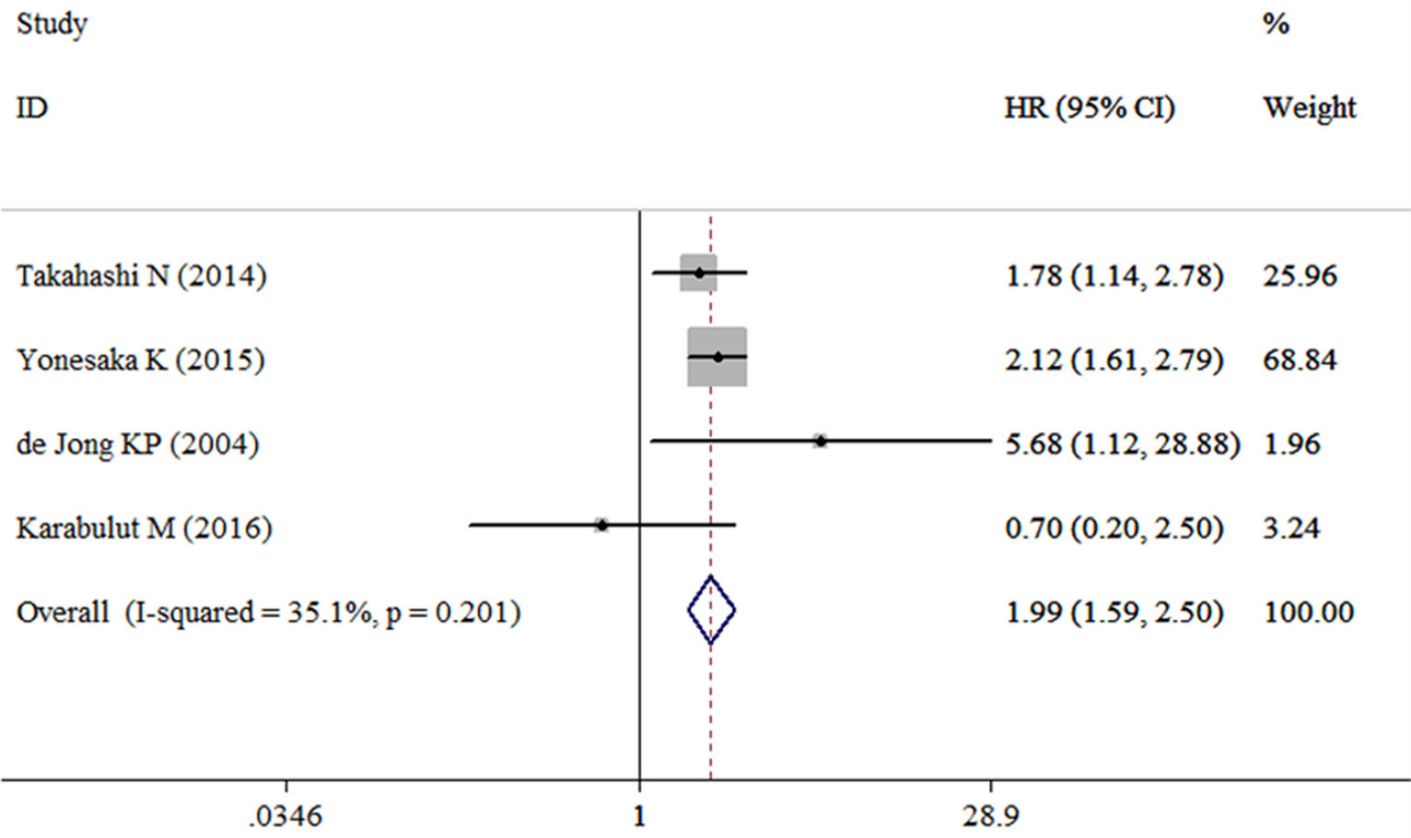
Forest plot evaluating the combined HR between HGF and DFS

**Figure 5:**
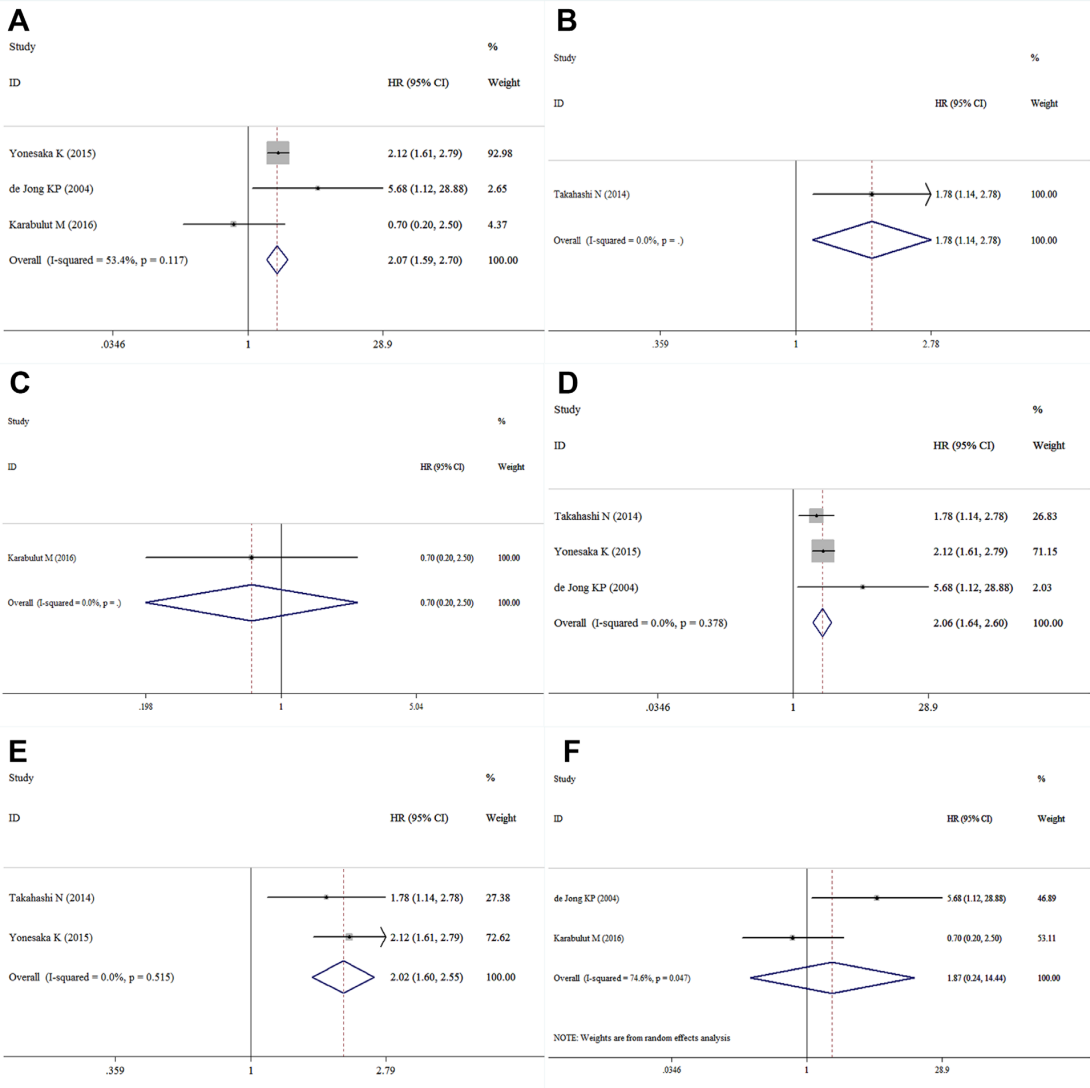
Forest plot of the subgroup analysis of HGF and DFS ((**A)**: univariate analysis; (**B)**: multivariate analysis; (**C**): Asian countries; (**D**): non-Asian countries; (**E**): I–IV CRC patients; (**F**): mCRC patients).

Four studies containing data of HGF and DFS in CRC patients were included in this research. The combined HR for the over-expression of HGF on DFS was 1.99 (95% CI: 1.59–2.50) (Table 2, Figure 4). Among these studies three with univariate analysis were homogenous (I^2^ = 53.4%, χ^2^ = 4.30, *P* = 0.117), and the combined HR was 2.07 (95% CI: 1.59–2.70) (Figure 5A). The HR for the studies with multivariate analysis was 1.78 (1.14–2.78) (Figure 5B). The combined HR for the patients with I-IV CRC and mCRC were 0.70 (95% CI: 0.20–2.50) (Figure 5C) and 2.06 (95% CI: 1.64–2.60) (Figure 5D), respectively. Among these four studies, two were conducted in Asian countries and the others were conducted outside from Asia. The combined HR for the Asian studies and the non-Asian studies were 2.02 (95% CI: 1.60–2.55) (Figure 5E) and 1.87 (95% CI: 0.24–14.44) (Figure 5F), respectively.

**Figure 6:**
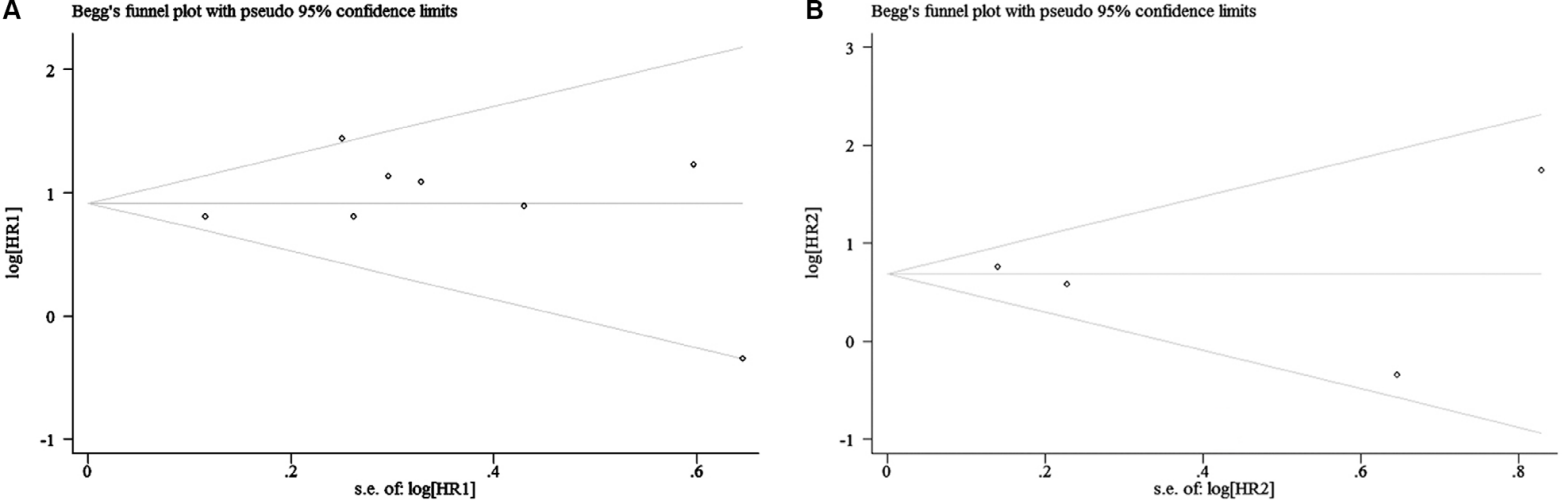
Funnel plot for all the included studies ((**A)**: OS; (**B**): DFS).

**Figure 7:**
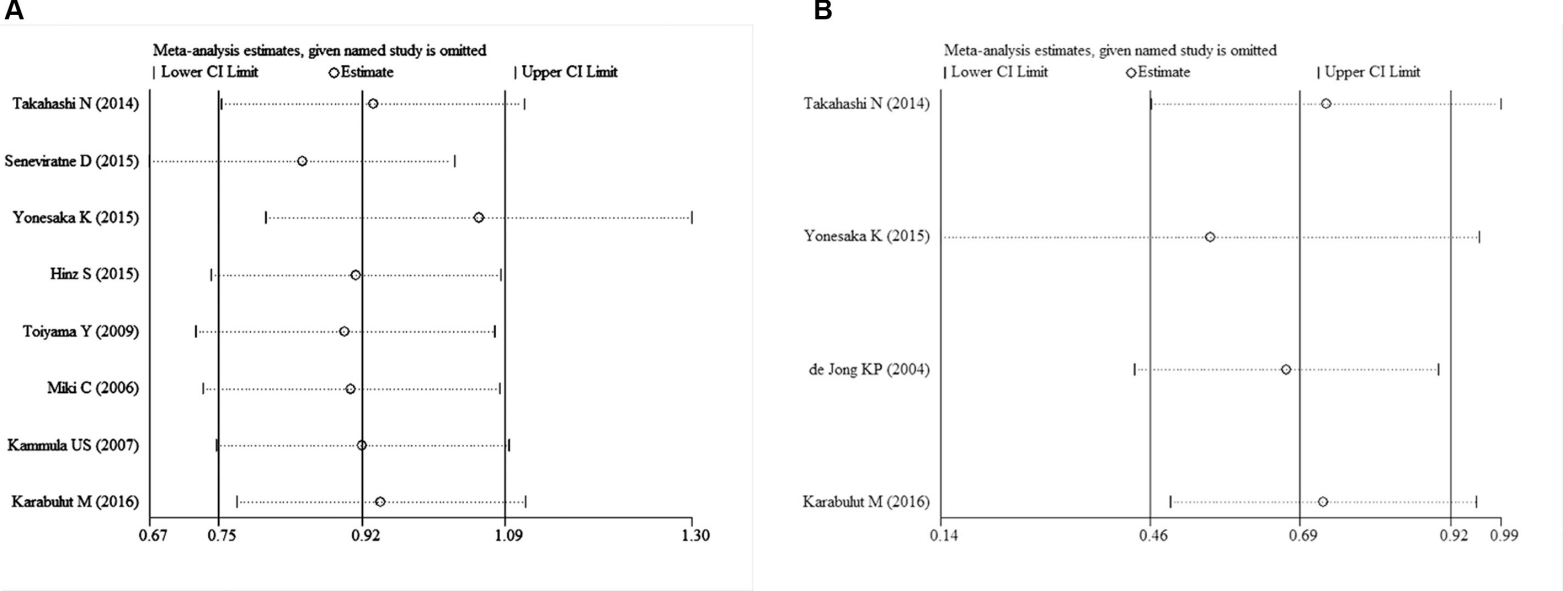
Sensitivity analysis for the included studies ((**A)**: OS; (**B)**: DFS).

**Figure 8:**
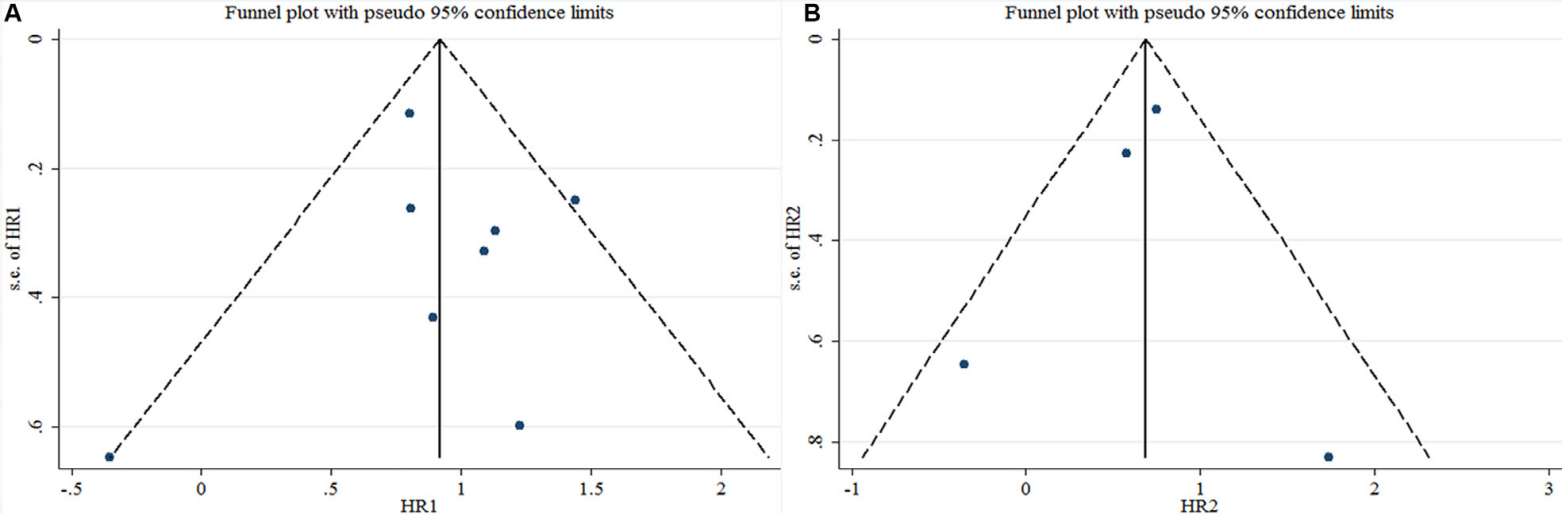
Funnel plots for the included studies ((**A**): OS; (**B**): DFS).

Begg's test was used to evaluate the publication bias. The results of Begg's test for both OS and DFS revealed no publication bias (P_OS_ = 0.536, P_DFS_ = 1.000) (Table 3, Figure 6A, and Figure 6B). A sensitivity analysis was then used to evaluate the influence of potential publication bias (Figure 7A, and Figure 7B). The funnel plots for publication bias were basically symmetric, which indicated the stability of the results (Figure 8A, and Figure 8B).

**Table 3:** Results of Begg's and Egger's tests

	Number of studies	Begg's test		Egger's test	
		*Z* value	*P*	*t* value	*P*
**Overall survival**					
**All**	8	0.62	0.536	0.23	0.827
Univariate analysis	4	0.34	0.734	0.02	0.987
Multivariate analysis	4	−0.34	1.000	0.27	0.814
**Patients**					
CRC	5	2.20	0.027	−6.97	0.006
mCRC	3	1.04	0.296	1.49	0.377
**Country**					
Asian	4	1.02	0.308	1.87	0.202
Non-Asian	4	1.02	0.308	−1.79	0.215
**Disease-free survival**					
**All**	4	−0.34	1.000	−0.24	0.832
Univariate analysis	3	−0.00	1.000	−0.13	0.916
Multivariate analysis	1	-	-	-	-
**Patients**					
CRC	1	-	-	-	-
mCRC	3	0.00	1.000	0.83	0.559

-: not applicable.

## DISCUSSION

This analysis provided evidence that over-expression of HGF could be a prognostic indicator in CRC. The over-expression of HGF contributed to lower OS and DFS in CRC patients via the targeting of HGF/MET signaling pathways. HGF, as the only known ligand for the MET proto-oncogene product of receptor tyrosine kinase, is converted into an active form that dimerizes and activates the MET receptor [18]. An active HGF/MET signal, which is involved in a number of malignancies, is associated with a poor prognosis and serves as an early predictor of further metastasis [19]. Toiyama reported that a high HGF level was associated with CRC development, lymphatic or distant invasive phenotypes and survival [14]. Therefore, poor prognosis is associated with a high HGF level. Similar results were also found in patients with gastric cancer [20], liver cancer [21], glioma [22], non-small cell lung cancer [23, 24], breast cancer [25, 26] and thyroid cancer [27, 28]. Additionally, two previous meta-analyses demonstrated that MET was associated with a poor prognosis of colorectal cancer [29] and gastric cancer [30].

Therefore, HGF-targeted therapy should be considered since the over-expression of HGF was confirmed to be the cause of poor prognosis in CRC. HGF inhibitors could potentially be effective in the inhibition of the HGF/MET axis in CRC patients with over-expression of HGF [12]. A randomized phase Ib/II trial indicated that anti-HGF monoclonal antibodies improved the overall remission rate and DFS in patients with mCRC [31]. Van Cutsem reported that combination therapy (including rilotumumab, an HGF inhibitor) showed promising activity compared with single-agent panitumumab in patients with chemo-refractory tumors (overall response rate was 31% versus 21%; PFS was 5.2 versus 3.7 months) [31]. Moreover, Yonesaka found that drug resistance to anti-EGFR antibody therapy caused by HGF might lead to a decreased OS [12]. Anti-EGFR antibody treatment was superior as a front-line therapy for patients with mCRC [32]. Hua demonstrated that an HGF inhibitor restored the sensitivity of the anti-EGFR antibody, which infers that HGF-targeted therapy might promote OS of CRC patients [33]. The data discussed above support the concept that the over-expression of HGF is a prognostic indicator in CRC, even though normal expression of HGF contributes to organ growth and development due to its capacity to aid in the regeneration of damaged liver, kidney and lung tissue and because of its protective function in the heart and brain [34].

A subgroup analysis showed that the results between different features were consistent, which means that the eligible studies were homogeneous. For example, the combined HR of the patients with stage I-IV CRC and the patients with mCRC were 3.08 and 2.26, respectively. The HRs of the stage I-IV CRC patients and the mCRC patients were not significantly different in terms of OS. The combined HR in the studies that used univariate analysis was 2.51, while that for the studies that used multivariate analysis was 2.65. The HRs of the CRC patients from non-Asian countries and those from Asian countries were not different. These results suggest that HGF had the same effect on the survival time of patients from different countries.

There are several limitations to this study: (1) The classification criteria for the over-expression and normal expression of HGF vary in the included studies, which might have been a confounding factor. However, the effects of different cut-off value would not be substantial. The reasons were as follow: The cut-off value of HGF ranged from 0.204 to 4.0 ng/ml in most of the eligible articles. Most of the eligible articles used scientific methods to classify HGF levels. The definition of the high expression of HGF is still controversial under current circumstance, similar to other genes in cancer, such as the carcino-embryonic antigen in CRC [35]. (2) Some HRs and their 95% CIs were calculated from the data extracted in survival curves (Kaplan-Meier curves), which might have caused subjective bias [36]. (3) Although we attempted to collect all relevant data on the patients included in these studies, some data might still be missing. For example, the treatment given to patients in some of the included studies was not reported in detail. The treatment played an important role in survival time, and different treatments might affect the prognosis of patients with over-expression of HGF. (4) The number of the included studies was small, especially in the study that reported DFS as its main outcome.

In conclusion, this meta-analysis demonstrated that HGF is associated with a poor prognosis (including poor OS and DFS) in patients with CRC. In this case, HGF may be a promising, new therapeutic target for CRC and may enable clinical practitioners to better predict patient prognosis through the detection of HGF levels in patients. However, this conclusion should be further confirmed by large-sample cohort studies.

## MATERIALS AND METHODS

### Search strategy

One reviewer (CXL) conducted a systematic literature search of PubMed, EMBASE, and the Cochrane Central Register of Controlled Trials databases from their inception until March 14th, 2016. The search included the following terms: colorectal cancer (including colon cancer and rectal cancer), hepatocyte growth factor (HGF, DFNB39, HPTA), and prognosis. We combined the term appropriately with MeSH terms. Details of the search strategies are shown in the Supplementary Appendix File 1. The meta-analysis was conducted in accordance with the PRISMA statement [37, 38]; the PRISMA 2009 Checklist is reported in the Supplementary Appendix File 2.

### Study selection

Studies that explored the correlation between HGF and the prognosis of patients with CRC were included. The eligible studies included cross-sectional studies, cohort studies, or randomized controlled trials. HR, its 95% CI, or the relevant information (such as a survival curve, namely Kaplan-Meier curves) was provided. The studies were also reported as full articles, which were published in English. The included patients were diagnosed with colorectal cancer (including colon cancer or rectal cancer), and patients were considered regardless of race, region, clinical stage, histological type, and treatment regimen. The prognostic outcomes included OS, DFS, and relapse-free survival (RFS). Studies published in abstract form were considered only if sufficient outcome data could be retrieved from the abstract or as a result of contact with the authors.

### Data extraction

Duplicate studies from different databases were identified, and the remaining abstracts were read for eligibility by two independent authors (LMG and MJL); the third author was involved in the reconciliation of studies with inconsistent results (CXL). The full texts of potentially eligible studies were retrieved and reviewed independently by two authors (HCY and ZQY). Any disagreements were recorded and resolved by consensus under the guidance of the third author (CXL).

The eligible studies were reviewed, and data were extracted independently by two authors (HCY and ZQY). The study information (the first author, the year of publication), study participants (the characteristics and sample sizes), characteristics of HGF, and prognostic outcomes (OS, DFS, and RFS) were extracted. If data from any of the above categories were not reported in the study, the item was recorded as “NR” (not reported).

### Data synthesis and analysis

The eligible studies were divided into the OS and DFS groups for the analysis. RFS was treated as DFS. The HGF value was classified as “over-expression” (high expression) or “low-expression” (normal expression).

The HR and its 95% CI were used to measure the effect of the over-expression of HGF on survival. If the HR and its 95% CI were given explicitly in the studies, we used the crude values. If these indexes were not given explicitly, they were calculated from the available numerical data (or survival curve, namely Kaplan-Meier curves) using the methods reported by Tierney et al. [39].

The heterogeneity of the individual HR was calculated using Chi-square tests. A heterogeneity test with inconsistency index statistic and Q statistic was also performed. If HRs among the studies were homogenous, a fixed effects model was used for analysis; if not, a random effects model was used. Subgroup analyses were performed according to different countries (Asian and non-Asian countries) and methods of analysis (univariate analysis, multivariate analysis). Some studies enrolled patients with metastatic colorectal cancer, while others enrolled patients with stage I-IV CRC. Subgroup analyses were also conducted according to the patient categories (I–IV CRC and mCRC).

A *P value* ≤ 0.05 was considered statistically significant. An observed HR >1 implied a worse prognosis in the case of HGF over-expression compared with the low expression of HGF. Publication bias was evaluated using Begg's test and funnel plots. All analyses were performed using STATA version 12.0.

## SUPPLEMENTARY MATERIALS FIGURES AND TABLES




