# Supplementary material for: Hepatocyte growth factor is a prognostic marker in patients with colorectal cancer: a meta-analysis
Source: Oncotarget. 2017 Feb 21;8(14):23459–69. doi: 10.18632/oncotarget.15589 (PMC5410318; doi:10.18632/oncotarget.15589)
Supplement: Supplementary file 1 [file oncotarget-08-23459-s001.pdf]

Hepatocyte growth factor is a prognostic marker in patients with colorectal cancer: a meta-analysis

Supplementary Materials

Supplementary Appendix File 1: The search strategy

All the searches were performed on March 14th 2016.

Pubmed

1. “colorectal Neoplasms” OR “colonic Neoplasms” OR “Rectal Neoplasms” [MeSH Terms] (160 836 articles)
2. “colorectal Neoplasms” OR “colorectal neoplasm” OR” colorectal tumors” OR “ colorectal tumor” OR “ colorectal carcinoma” OR “colorectal carcinomas” OR “colorectal cancer “OR “colorectal cancers” OR “colonic Neoplasms” OR “colonic neoplasm” OR” colonic tumors” OR “ colonic tumor” OR “ colonic carcinoma” OR “colonic carcinomas” OR “colonic cancer “OR “colonic cancers” OR “rectal Neoplasms” OR “rectal neoplasm” OR” rectal tumors” OR “ rectal tumor” OR “ rectal carcinoma” OR “rectal carcinomas” OR “rectal cancer “OR “rectal cancers” [Title/Abstract] (175 731 articles)
3. #1 OR #2 (184 867 articles)
4. “hepatocyte growth factor” OR HGF OR DFNB39 OR F-TCFB OR HPTA [Title/Abstract] (12 029 articles)
5. Prognosis OR Prognoses OR Prognostic OR predictive OR biomarker OR marker OR Survival OR Survive OR Cox OR Logrank OR Kaplan-Meier [Title/Abstract] (3 499 901 articles)
6. #7 AND #8 AND #9 (81 articles)

Embase

1. “colorectal Neoplasms”/exp OR “colonic Neoplasms”/exp OR “Rectal Neoplasms”/exp (275, 151 articles)
2. “hepatocyte growth factor” OR HGF OR DFNB39 OR F-TCFB OR HPTA (14,806 articles)
3. Prognosis OR Prognoses OR Prognostic OR predictive OR biomarker OR marker OR Survival OR Survive OR Cox OR Logrank OR Kaplan-Meier (2,685,955 articles)
4. #1 AND #2 AND #3 (232 articles)

Cochrane

1. “colorectal Neoplasms” OR “colonic Neoplasms” OR “Rectal Neoplasms” (6,052 articles)
2. “hepatocyte growth factor” OR HGF OR DFNB39 OR F-TCFB OR HPTA (212 articles)
3. Prognosis OR Prognoses OR Prognostic OR predictive OR biomarker OR marker OR Survival OR Survive OR Cox OR Logrank OR Kaplan-Meier (103,210 articles)
4. #1 AND #2 AND #3 (2 articles)

Supplementary Appendix File 2: PRISMA 2009 Checklist. See Supplementary_Appendix_File_2
